# Introductory Lecture to the General Course of Mechanical Dentistry

**Published:** 1857-12

**Authors:** J. Richardson


### DENTAL REGISTER OF THE WEST.

VOL. XI.—DECEMBER, 1857.—NO. 2.

### Original Essays and Communications.

### INTRODUCTORY LECTURE TO THE GENERAL COURSE ON MECHANICAL DENTISTRY,

Delivered in the Ohio College of Dental Surgery.

BY J. RICHARDSON, D. D. S.

GENTLEMEN:—Before proceeding to the main topic of this introductory, I desire to preface it with a few brief personal remarks.

Through the kindness and partiality of the Stockholders and Board of Trustees of the school with which I stand connected, I have been assigned the important and responsible duties connected with the Chair of Mechanical Dentistry. I should confess myself ungrateful and disingenuous, not to acknowledge myself flattered by this testimonial of confidence or the compliment implied in the appointment. It is but just, however, to myself to state that I did not in any way seek for or solicit the place. A friendly and earnest solicitude for the permanent welfare and continued usefulness of the school which I take great pride in acknowledging as my Alma Mater, would have made it a matter of equal and greater pleasure to me to have seen some one placed here, who, better than myself, might have supplied the wants of the student, and who might have given to the friends of Collegiate instruction, here and elsewhere, better assurances of aptitude and qualification. But the position having been tendered and accepted, I now stand fairly committed to a discharge of the duties imposed by it; and I can imagine no obligations higher or more imperative in their nature than those imposed by the circumstances of my election. I can not fail to recognize in this generous and unsolicited tender of confidence and trust, the strongest possible incentive to an unqualified and cordial application of my best powers to the duties of the chair. Whatever resources I may possess shall be entirely at your service, and may I not expect in return, gentlemen, that *you* will as cordially extend to me, not only a respectful attention, but that degree of indulgence which *inexperience* in a new adventure may rightfully exact. In the performance of an untried duty, and fortified by no great amount of self-assurance, I shall probably furnish you frequent occasion for the exercise of that peculiarly Christian virtue,—*charity.*

Our relation is one of the most intimate, as it should be one of the most agreeable, nature. Association seldom weaves stronger or more enduring ties than those that bind the faithful teacher to his class or the faithful student to his preceptor. It is not a mere temporary or transient feeling of interest and respect by courtesy, or of short-lived gratitude and attachment that ends with the immediate compact. But while these sentiments widen and deepen during the immediate relation, we should aim to carry them with us into the wide world, there to strengthen and develop into matured and reciprocal friendships amidst the cares and distractions of active professional life. We hope ultimately to become members of the same great brotherhood; inspired by the same hopes, depressed by the same apprehensions and discouragements, allied by common interests, and cheered by the same prospects that tend to enlarge the usefulness, to enhance the character, and promote the fortunes of our common calling.

It is a relation, likewise, that imposes mutual obligations. He who voluntarily assumes the functions of a public instructor, does so with the implied understanding that the treatment of the subjects connected with his particular department shall fully answer the demands of the most rigid exaction on the part of the student. To satisfy these requirements demands thorough qualification, a large expenditure of time, much and close application, and no inconsiderable sacrifice of private interests. It is but reasonable under these circumstances, therefore, that he may ask of his class, as a counter-obligation, a deportment in all respects consistent with the requirements of social life, an earnest and respectful attention to his ministrations, and a reasonable diligence in the pursuit of their studies.

But I need not amplify upon this topic of reciprocal duties and obligations. You will at once recognize and appreciate them. I have a confident assurance that I may rely upon your intelligence and good-nature, to indulge me in whatever imperfections may characterize a first trial in a new field of labor. It is not for me to anticipate or predict the probable success of my course. Whatever an honest purpose and ambition to serve you may accomplish, shall be done, in which I shall endeavor to *command*, if I may not *solicit*, your approbation.

Without occupying your attention further with these personal reflections, I desire for a short time to direct your attention to the main subject of this introductory, in which we propose to consider

*The conditions of professional success considered in relation to the practice of Dental Mechanism.*

In this day and age, when mankind are proverbially prone to consider that *success* which proves to be merely personally *advantageous*, it may be proper for us to introduce our subject by a brief analysis of the term. And we shall, perhaps, more clearly understand its true signification by first pointing out some of the manifest perversions, which, in popular estimation, attach to this condition in life.

For example, in *politics*, a man with talents scarcely respectable, and wholly innocent of any moral qualifications, attains to political preferment. Every unmanly expedient and disgraceful practice known to the calendar of corrupt cliques, has been exhausted to accomplish a dishonorable triumph. Now is this in any just sense, *success?* Yet the rabble applaud, and friends hasten to congratulate him, while the world, so far as it may know or care for the event, tacitly admits his cleverness and advantage. To a mind imbued with a just conception of the true import of the term, the mere attainment of *position* by such instrumentalities, is a reproach, and the so-called success, contrasted with a true representative of the idea, must be deemed by all honorable men a miserable failure. Whatever apparent prosperity, popularity or favor may characterize the career of such a man, rest assured that neither the distinction of an *Honorable* title or the ephemeral outbursts of popular applause can redeem his name from certain and deserved oblivion.

The same is equally true in the practice of the legal profession. The advocate who, by every available species of legal quibbling and unscrupulous management, carries his cause in plain violation of all equitable principles, will be deemed by the world generally more *successful* than the conscientious lawyer who scorns such unprofessional tricks. Is the world right in its estimate ? The one achieves a victory at the expense of a moral blemish which no conceivable reputation for shrewdness or tact can either extenuate or wash out. The turpitude of the act is as apparent to our moral consciousness as the brand of guilt on Cain's forehead. Success in its true acceptation rests with him who, though foiled by superior arts, stands vindicated in his own soul against all imputations of connivance at wrong or complicity therein. The one utterly fails in the midst of a supposed triumph; the other is strengthened in the hour of apparent defeat by a heroic and self-sacrificing loyalty to his own manhood.

Again, our elder sister, Medicine, with all her virtues, is prolific in illustrations of this false and perverted notion of professional success. The self-assured, adroit, and unscrupulous interpreter of mankind,finds abundant material with which to crowd his visiting list. Patronage flows in upon him through practices that would irretrievably disgrace him in the Academy. He may be quite guiltless of ever having conceived a single useful or original idea in his life, or of having in any creditable degree comprehended the great functions of his calling. He may have been guilty of confounding flea bites with the eruption of small-pox; of applying plasters to the shoulder in inflammation of the liver; of ligating the common carotids in apoplexy; blundering with the unconscious knife into anurismal sacs in search of abscesses;—of doing anything and everything that is particularly unskillful and outlandish, and yet he may continue to feed and fatten on public credulity, while the same victimized public looks complacently on, and exclaims, "He is *very* successful."

We might extend this view into all the departments of human industry and enterprise, and in its practical manifestations we should find that *success*, in the minds of most men, is comprehended in the narrow and selfish, but great and overshadowing idea of *self-aggrandizement.*

But we must bring these remarks down to a more immediate and direct application to the subject under consideration. It is scarcely necessary to remind you that the practice of Dentistry forms no exception to the prevailing tendency to confound professional success with gratified self-interest; that practitioners of our art are not all exemplary gentlemen, or that the arts of charlatanry sometimes go hand in hand with increasing patronage. The facts hinted are patent to you all, and need no affirmation from us. Every community furnishes its quota of professional traffickers, who cater to the popular estimate of professional worth, by means of clap-trap advertisements, boastful private pretensions, and the huckstering exhibition of wares at cattle shows.

Whatever may be the rewards and emoluments of these unworthy, mercenary, and self-imposed prostitutions of legitimate dentistry, they can not either by courtesy or concession be considered as having, in the remotest degree, any relationship with professional success. The term has a higher signification, and over-rides all considerations of *mere* profit. It implies a standard of attainments that subordinates all questions of mere selfish policy or pecuniary advantage to the higher claims of honorable distinction, of pride of character, of irreproachable personal integrity, and an incorruptible fidelity to the paramount interests of the profession. The aims contemplated in this standard, to our apprehension, are those only which can give a rational assurance of success, and it matters little whether an honest endeavor in their attainment is found practically associated with preëminent abilities or inferior endowments; with opulence or indigence, the faithful fulfillment of their implied obligations to the extent of each separate capacity, is a *success.*

It is our purpose to indicate the means by which this standard may be attained, or in other words, to consider the *conditions* of professional success. We propose also to discuss them in relation to the practice of *Mechanical Dentistry*, as distinguished from strictly Operative or Surgical practice.

We shall be excused by all intelligent and candid minds from any attempt directly to vindicate the legitimate practice of this important speciality from the ill-natured contempt affected by certain fastidious, exclusive, kid-gloved gentry in the profession, whose overcharged sensibilities instinctively shrink from all contact with *undignified* labor. Uninformed of its true scope and purposes, and practically unacquainted with its details, and withal incompetent, perhaps, through natural disqualifications, to attain to any respectable degree of proficiency in its practice, to disparage and depreciate, by injurious and offensive comparisons, is felt to be the only convenient, if not creditable, means of escape from self-accusation.

In general terms, it may be stated that no one is fully com petent to practice dental mechanism who is not familiarly acquainted with the entire curriculum of studies, as generally taught in every properly organized dental school. This comprehends a knowledge of Chemistry, Metallurgy, Anatomy, Physiology, Materia Medica, and Therapeutics. Natural or Mechanical Philosophy may also be considered an indispensable adjunct. We shall hardly fail to recognize in these requirements the most satisfactory defense of this branch of dental science from the ill-judged imputations of mere *finger-smithing.* While it may be true that the veriest handicraftsman may familiarize himself with the routine manipulations of the laboratory, and demonstrate his title to skillfulness in the mere construction of a piece of plate work, it is equally true that the skill and judgment necessary to conduct an operation in all its diversified bearings and stages, from its first inception to its final and successful adjustment in the mouth, demands something more than crude and unassisted mechanism. These requirements we shall consider in the order just enumerated, without intending to discuss them fully.

*Chemistry,* in its extended range of application to the necessities of the mechanical dentist, becomes a subject of great practical importance. It suggests so many modifications of practice in connection with the mouth, and is so immediately concerned in many of the more important processes of the laboratory, that to be ignorant of its principles is to blunder and fail continually, just where it is important to do neither.

Organic or Physiological Chemistry, by analysis, makes us acquainted with the ultimate composition of the different structures of the mouth, the condition of its secretions in health and disease, and the various chemical actions that take place in this cavity under favoring conditions. The application of these facts is obvious. We will offer a single example in illustration. We are called upon to adapt an artificial piece to a mouth the secretions of which are highly impregnated with free acids. Chemistry determines this fact by appropriate tests. The insertion of destructible material into this mouth without some preparatory treatment, would, to say the least, be injudicious. The vitiated fluids would act to some extent upon the plate, and this, with its alloys furnishing favorable conditions for galvanic action, would react upon the secretions, liberating increased quantities of the destructive element. Thus the patient would not only be subjected to continued discomfort, but a positive evil would unquestionably be inflicted. Chemistry is not only *detective*, but suggests the means by which these consequences may at least be mitigated. The educated practitioner is not only forewarned, but forearmed. His duties do not rest or end with a knowledge of the predictions of science; but if he proceeds in good faith to his patient, he begins by inquiring into the cause or causes of this abnormal condition. Chemistry teaches him the harmless character of these secretions in health, and his first care is to detect the influences concerned in their perversion. If it arises from decomposing foreign substances, the offending cause is removed. If from gastric derangement, functional disturbance of the glandular system, local inflammation, or from any of the multiform constitutional conditions associating with them these changes in the healthy character of the fluids, Chemistry may still, in aid of general medicine, be invoked to restrain with neutralizing agents. If the cause can not be reached and removed, Chemistry indicates the means and material that will most effectually resist the operation of these offending agents.

This presents but a partial view of the application of Organic Chemistry to the practical duties of the professional artisan. It is enough, perhaps, to indicate its general bearing.

*Inorgunic Chemistry*, again, has yet a still wider range of application in the laboratory. It would be inconsistent with our limits to particularize. The success of the means, and the behavior of the materials employed, may be certainly predicted, and the operator spared much annoyance, mortification, and positive loss, by an acquaintance with the principles of this important science. *Experience* has the credit of being an efficient teacher; but it is rather unphilosophical, I take it, to subject one's-self, without sufficient cause, to the usual penalties consequent upon this species of acquisition.

There is another aspect of this subject of a prospective character that has in it something of interest, as well as of promise, to the aspiring student. Whatever advancement in the dental art may characterize the next quarter of a century, it is safe to predict that it will chiefly grow out of the unappropriated or undeveloped resources of chemical science. The field in this direction, therefore, furnishing such ample prospects of distinction, will give to your opportunities all the force of an obligation to confer in some important respect or other, lasting benefits on mankind and the profession, to say nothing of yourselves. It may be made to enter as an element into your chances of professional success.

A thorough acquaintance with *Metallurgy*, in connection with its kindred science, Chemistry, is so plainly a condition of success in the intelligent conduction of the various processes of the laboratory, that it is almost needless to urge its importance. He has but illy fulfilled his obligations who rests contented with the simple knowledge of those more commonplace phenomena that address themselves obviously and directly to the senses, as, that certain substances when subjected to sufficient heat, pass from the solid into the fluid state; that an ingot of gold may be rolled into No. 26 plate; that zinc is more resistent under the hammer than lead, etc., etc. Any tyro may know these things, and much more of the sort; but a bare knowledge of these sensible properties, and the like, falls far short of the just demands of this important study. The practised and accomplished dental artificer presses a higher order of facts and principles into his service. He makes himself acquainted with the power and influences of heat in all of its complex and diversified actions upon the metals and other materials of the shop; the selection and management of fuel; the best methods of producing and regulating desirable degrees of heat, and the power of this agent in the production of certain chemical and physical changes upon different substances subjected to its influence. He inquires into the origin, mode of procurement, commercial history, treatment, physical characteristics, and behavior of the metals he employs; their combinations with each other, and the nature and properties of the resulting alloys; the action of re-agents and other means employed in the separation and purification of the latter; rules employed in determining the exact composition of his plate and alloys; and divers other matters of interest and subjects of great moment that can not be ignored or dispensed with, without an almost certain chance of failure in the general management of the materials and processes employed.

We pass now from these subjects of more immediate and pressing application to the exigencies of mechanical practice, to a consideration of other acquisitions of not much less importance, perhaps, but of less *apparent* utility to the mechanical dentist. I do not use this latter term in the restricted sense of a mere plate-worker. His office clearly comprehends as well, the extraction of teeth, the surgical and medical treatment of the mouth, the skillful adjustment of the artificial piece therein, and the rectification of any unhealthy conditions that may thereafter become complicated with the case. In other words, all these processes are essentially and practically a *unit*, and can not be divested of this character without detracting from the proper and natural functions of the operator. These claims conceded, we come first in order to a consideration of

*Anatomy.* The necessity of a correct anatomical knowledge of the different structures of the mouth to the mechanical operator will hardly be questioned. The physical characteristics of the teeth, is a study in itself. If he would aspire to a successful imitation of nature in his methods of reparation, he must familiarize himself with her natural and customary aspects. He must study the forms, size, coloring, relations and antagonisms of the natural organs. Just in proportion as he shall succeed in reproducing these original forms, conditions and appearances, will he excel in one of the legitimate purposes of his art. In this particular at least, we may, without offending against correct morals or the exactions of professional good faith, indulge him in the practice of deception.

The structural anatomy of the teeth also, especially in connexion with plates attached by clasps, must be duly considered. There are certain structural features, that very certainly indicate or contra-indicate the fitness of this or that tooth for the purposes of support. We can not disregard such indications without hazard to our own reputation, and risk of positive injury to the patient. At best, this method of substitution, under the most favorable circumstances, and surrounded by every safeguard that skill or ingenuity can devise, is not free from serious objections. This circumstance in itself imposes additional obligations upon us to mitigate, by intelligent discrimination, what seems to be the infliction of a *necessary evil.*

Again, the proper preparation of the mouth involves some knowledge of anatomy. Whatever dissections or excissions may be indicated to facilitate the operations of nature in the removal of superfluous structures, should be directed by enlightened skill, if we would escape the odium of inexcusable butchery. When, after this preparatory treatment, the parts are committed to the absorbents to finish the work, the critical study of the anatomy of the mouth will better enable us to determine the period of its completion. By this means the mortification incident to failure in such cases, and much subsequent annoyance and trouble, both to operator and patient, may be spared. It will also enable us, with greater accuracy, to determine the proper dimensions of our plate, allowing for the customary play of muscles, the avoidance of undue contact with glands and the intolerance of the soft parts to undue pressure and irritation.

In that important branch of dental prosthesis relating to certain appliances known as *obturators*, an intimate knowledge of all the parts associated with the mouth, is an indispedsible pre-requisite of success in their application. Palatine fissures or cleft-palate, demands an enlightened judgment to determine upon the best plan of remedying the defect by artificial means, as well as consumate skill in the mechanical execution of the fixtures. The former can not well be comprehended without a correct understanding of the structure, form, and functions of the parts implicated, and without this precedent knowledge, the purpose contemplated by the mechanical appliance, by consequence, fails.

In the correction of irregularities in the arrangement of the natural teeth, every appliance designed to remedy these defects, must have a very direct reference to existing anatomical conditions. That we may determine beforehand with greater accuracy, the relative value of this or that mode of procedure, it will serve us to know what particular anatomical conformations may resist or favor the application of forces. The comfortable and efficient adaptation of fixtures, will also depend very much upon an acquaintance with the anatomical peculiarities of each particular case. This consideration can not be overlooked. But we must pass to

*Physiology, Pathology, Materia Medica and Therapeutics.* These different branches of medical science are so closely associated in their practical bearings, that we propose to consider them in immediate connexion. Physiology, relating as it does to those phenomena that manifest themselves in connexion with the living organism during *health* or *normal life*, becomes a study worthy of all men, whether professional or otherwise, for it is the science of *human existence,—the philosophy of life.* To the physician and dentist, it is the *sine qua non* of all rational and legitimate practice. Intimately associated as are the different organs of the mouth with the great functions of digestion, circulation, nutrition, respiration, nervous influence, etc., it becomes a matter of the first moment, that we should acquaint ourselves with the laws and conditions that determine the healthful performance of these functions. The wide range also of nervous phenomena, growing out of the mutual, intimate and mysterious relations of the several organs of the body, are continually susceptible of almost infinate modifications of irregular or diseased manifestation from the multiplied influences of disturbing causes. We should understand clearly the nature and extent of these relations, and should as fully comprehend the nature of those *changes* also, that mark the transition stage from health to disease. Our limits will only permit us to make but a brief and single application of these remarks in illustration. You are called upon, as you often will be, to treat some form of inflammation, in some tissue of the mouth. To do so intelligently, you can not content yourselves with a knowledge of its more common signs, as heat, swelling, redness and pain, and thereupon pronouncing it an *inflammation*, proceed to treat it as you would treat a boil or a bunion. Any unlettered mountebank may do the same, and do it as well. We should study its diversified phases, enquire into its origin and causes,—determine its character, whether it is simple and uncomplicated, or ulcerative; suppurative or gangrenous; specific, malignant or benign. We should be able to determine whether it is strictly idiopathic or local, or if it is but the open manifestation of some constitutional perversion. It is such qualifications that distinguish the educated practitioner from the mere pretender. In the treatment of the various affections of the mouth, you will find ample scope and opportunity for the exercise of the nicest judgment and discrimination. A large proportion of all cases coming to you requiring the insertion of artifical pieces, will call for more or less of preparatory medical or surgical treatment. Your success will depend in great part upon your skill in this important respect.

Materia Medica and Therapeutics are indispensible studies. The former will instruct you in a knowledge of the nature and properties of remedial agents, while the latter points out their mode of operation, their application to particular forms of disease, the circumstances modifying their action, etc. In brief then, all these studies in their practical relations to each other are inseparable. As we shall become acquainted with the healthy functions of the body, and are enabled to recognise and comprehend the changes that constitute its diseases, and have familiarized ourselves with the nature, properties and power of remedial agents, and have studied well their therapeutical action in health and disease, just to that extent shall we qualify ourselves for the duties of our profession, and arm ourselves with instrumentalities that can not fail ultimately to command success.

The application of the principles of *mechanical philosophy* to the purposes of the mechanical dentist, has reference more especially to the construction of the various fixtures and appliances that tend to facilitate him in the execution of his peculiar work. These appliances are continually enlarging in their range of application, and demand corresponding modifications, improvements and augmentations. To meet these growing wants, calls for the exercise of inventive genius but inventive genius alone will scarcely achieve any results of a positive practical nature, without some acquaintance with those natural laws necessary to direct it successfully in the pursuit of a particular end. In proportion as methods and processes multiply in our art will the necessity of increased facilities be felt. These wants must be mainly supplied by those engaged immediately in practice. None understand so well their necessities, or are so competent to meet the exigencies of daily practice. Natural or mechanical philosophy, therefore, becomes plainly a study proper and indispensable, if we would, by cultivating a spirit of self-reliance in the profession, aim to develope in the most eminent degree, the highest resources of the art.

In concluding this subject we desire to advert briefly to a single other qualification which we can not but esteem as indispensable to complete success in the practice of dental prosthesis, which is, that every operator whose function it is to restore the loss of the natural teeth should be an *artist.* I use this term in its higher sense as allied to those attributes that excel in the beautiful creations of music, poetry and sculpture. There is in every human face, a peculiar combination and play of features, that gives to it a determinate and distinctive character, distinguishing it from all other faces, and constituting *individuality.* Not a single feature in this face may be in the least degree marred, without altering or impairing its predominant and characteristic expression. Who has not at some time or other, experienced an instinctive and involuntary feeling of repugnance, on beholding some of those occasional deformities of the face, resulting either from disease, accident or congenital defect. One of the most prevailing deformities of facial expression is found associated with the partial or entire loss of the dental organs. It is not by any means the result of that natural decay of the organism consequent upon advanced age. The stripling not out of his teens comes to us with an unnatural aspect asking our aid, and manhood, scarce in its prime, with a jargon of inarticulate words and distorted visage, asks to be seated for a full set. The arch school-girl not yet out of short gowns, comes lisping through gaping incisors with a "pleathe thir, put me in a tooth," while the maiden just ripening into womanhood, with the flush of health upon her brow and cheeks, and the light of loveliness in her eye, comes, a piteous spectacle of toothless jaws and sunken lips, to implore of art the restitution of those gems, without which all other charms of person are but mockeries. And thus it is with the middle-aged without number. It will be our peculiar office to assuage the natural grief and pain of such afflictions, to relieve and mitigate these deformities, to soften repulsiveness, to dissipate the anxious solicitude of affection and friendship, by restoring lost expression, and to bring back to the hapless sufferer the pleasing and familiar voice that fell so sweetly and unbroken upon the ear in times past. To insure, in the highest degree, success in these humane purposes, involves the study of the harmony of artificial substitutes in form, size, coloring, arrangement, antagonism, etc., with the collective and characteristic features of each separate countenance. As before remarked, every one has a peculiar and distinctive personality. While it is prominently the office of the strictly operative dentist to *preserve* this personality unimpaired, it is equally the province of the dental artificer, by the exercise of a yet higher order of skill, to *restore* this lost harmony to its natural and customary aspect. This requires the nicest artistic judgment and discrimination. Teeth that would add attractiveness to beauty might caricature homeliness, and render natural defects of person more glaring by open contrast; and organs that might appear seemly in the mouth of a burly, broad-faced, dusky-hued waiting-maid, would, in the mouth of a symmetrically-formed, fair-skinned, blue-eyed belle, disgrace us forever. There are distinctive physical characteristics in every face that require a harmonious denture, if we would be true to nature. The subject will be more fully treated of in its proper place. We shall only add that the selection and arrangement of artificial teeth, calls for the most accurate and cultivated perception of the prevailing association of certain features, with a particular temperament, habit of body, age, size, sex, facial conformation, figure, etc., and that this fact is so certain in its indications that it can not be generally disregarded without offending against good taste, outraging nature, or incurring the odium of unskillfulness. And yet to disregard it is common in the profession; a fault attributable either to ignorance, carelessness or the hurry incident to an overgrown practice. We have in our mind's eye, operators in full practice, the artificial teeth of whose patients, more certainly indicate their origin than Wedgewood wares, or Barlow knives. They are hibitually characterized by a most disagreeable and oppressive *sameness*. The family likeness running through them at once excludes all doubt as to relationship or paternity. They never lose their identity, but may be known by whom they were begotten almost as far as a rifle will carry. However well or highly finished such pieces may be, they can hardly be considered *artistic* in any just sense of the term.

But we have already wearied your patience and exceeded the limits we had prescribed for our subject. When we meet you again it shall be to enter more directly and particularly into a discussion of the principles, the practical application of which, to the mechanical appliances of our art, we trust shall lead to professional success in the future practice of your profession.

